# Proportion of clinical holistic responders in patients with persistent spinal pain syndrome type II treated by subthreshold spinal cord stimulation compared to best medical treatment: a study protocol for a multicentric randomised controlled trial (TRADITION)

**DOI:** 10.1186/s13063-023-07140-3

**Published:** 2023-02-20

**Authors:** Lisa Goudman, Koen Putman, Leen Van Doorslaer, Maxime Billot, Manuel Roulaud, Philippe Rigoard, TRADITION consortium, Bart Billet, Bart Bryon, Mark Plazier, Vincent Raymaekers, Johan Vangeneugden, Maarten Moens

**Affiliations:** 1 STIMULUS Research Group, Vrije Universiteit Brussel, Laarbeeklaan 103, 1090 Brussels, Belgium; 2 Department of Neurosurgery, Universitair Ziekenhuis Brussel, Laarbeeklaan 101, 1090 Brussels, Belgium; 3 Center for Neurosciences (C4N), Vrije Universiteit Brussel, Laarbeeklaan 103, 1090 Brussels, Belgium; 4 Department of Physiotherapy, Pain in Motion Research Group (PAIN), Human Physiology and Anatomy, Faculty of Physical Education & Physiotherapy, Vrije Universiteit Brussel, Laarbeeklaan 103, 1090 Brussels, Belgium; 5 Research Foundation Flanders (FWO), Egmontstraat 5, 1000 Brussels, Belgium; 6 Department of Public Health (GEWE), Faculty of Medicine and Pharmacy, Vrije Universiteit Brussel, Interuniversity Centre for Health Economics Research (I-CHER), Laarbeeklaan 103, 1090 Brussels, Belgium; 7 PRISMATICS Lab (Predictive Research in Spine/Neuromodulation Management and Thoracic Innovation/Cardiac Surgery), Poitiers University Hospital, 86021 Poitiers, France; 8 Department of Spine Surgery & Neuromodulation, Poitiers University Hospital, 86021 Poitiers, France; 9 Pprime Institute UPR 3346, CNRS, ISAE-ENSMA, University of Poitiers, 86360 Chasseneuil-du-Poitou, France; 10 Department of Radiology, Universitair Ziekenhuis Brussel, Laarbeeklaan 101, 1090 Brussels, Belgium

**Keywords:** Pain management, Chronic pain, Failed back surgery syndrome, Neuromodulation, Composite measure, Randomised controlled trial

## Abstract

**Background:**

Integrating information on bodily functions, pain intensity and quality of life into one composite measure of a holistic responder has recently been proposed as a useful method to evaluate treatment efficacy of spinal cord stimulation (SCS) in patients with therapy-refractory persistent spinal pain syndrome type II (PSPS-T2). Previous studies already demonstrated the efficacy of standard SCS over best medical treatment (BMT) and the superiority of new subthreshold (i.e. paresthesia free) SCS paradigms compared to standard SCS. Nevertheless, the efficacy of subthreshold SCS compared to BMT has not yet been investigated in patients with PSPS-T2, neither with unidimensional outcomes nor with a composite measure. The current objective is to examine whether subthreshold SCS, compared to BMT, provided to patients with PSPS-T2 results in a different proportion of clinical holistic responders (as composite measure) at 6 months.

**Methods:**

A two-arm multicentre randomised controlled trial will be conducted whereby 114 patients will be randomised (1:1) to (a) BMT or (b) paresthesia-free SCS. After a follow-up period of 6 months (primary time endpoint), patients receive the opportunity to cross over towards the other treatment group. The primary outcome is the proportion of clinical holistic responders at 6 months (i.e. a composite measure of pain intensity, medication, disability, health-related quality of life and patient satisfaction). The secondary outcomes are work status, self-management, anxiety, depression and healthcare expenditure.

**Discussion:**

Within the TRADITION project, we propose to shift the focus from a unidimensional outcome measure towards a composite measure as primary outcome measure to evaluate the efficacy of currently used subthreshold SCS paradigms. The lack of methodologically rigorous trials exploring the clinical efficacy and socio-economic consequences of subthreshold SCS paradigms is pressing, especially in light of the growing burden of PSPS-T2 on the society.

**Trial registration:**

ClinicalTrials.gov NCT05169047. Registered on December 23, 2021

## Administrative information

Note: the numbers in curly brackets in this protocol refer to SPIRIT checklist item numbers. The order of the items has been modified to group similar items (see http://www.equator-network.org/reporting-guidelines/spirit-2013-statement-defining-standard-protocol-items-for-clinical-trials/).

**Table:** 

Title {1}	Proportion of clinical holistic responders in patients with Persistent Spinal Pain Syndrome Type II treated by Subthreshold Spinal Cord Stimulation compared to Best Medical Treatment: a study protocol for a multicentric randomised controlled trial (TRADITION)
Trial registration {2a and 2b}	ClinicalTrials.gov NCT05169047. Registered on December 23, 2021
Protocol version {3}	Protocol Version 2, December 2021.
Funding {4}	This study is funded by Research Foundation Flanders (FWO), Belgium (project number 12ZF622N).
Author details {5a}	Lisa Goudman^1−5^, Koen Putman^6^, Leen Van Doorslaer^1^, Maxime Billot^7^, Manuel Roulaud^7^, Philippe Rigoard^7−9^,TRADITION consortium, Maarten Moens^1−4,10^ ^1^STIMULUS research group, Vrije Universiteit Brussel, Laarbeeklaan 103, 1090 Brussels, Belgium. ^2^Department of Neurosurgery, Universitair Ziekenhuis Brussel, Laarbeeklaan 101, 1090 Brussels, Belgium. ^3^Center for Neurosciences (C4N), Vrije Universiteit Brussel, Laarbeeklaan 103, 1090 Brussels, Belgium. ^4^Pain in Motion Research Group (PAIN), Department of Physiotherapy, Human Physiology and Anatomy, Faculty of Physical Education & Physiotherapy, Vrije Universiteit Brussel, Laarbeeklaan 103, 1090 Brussels, Belgium, www.paininmotion.be. ^5^Research Foundation Flanders (FWO), Egmontstraat 5, 1000 Brussel, Belgium. ^6^Interuniversity Centre for Health Economics Research (I-CHER), Department of Public Health (GEWE), Faculty of Medicine and Pharmacy, Vrije Universiteit Brussel, Laarbeeklaan 103, 1090 Brussels, Belgium. ^7^PRISMATICS Lab (Predictive Research in Spine/Neuromodulation Management and Thoracic Innovation/Cardiac Surgery), Poitiers University Hospital, 86,021 Poitiers, France. ^8^Department of Spine Surgery & Neuromodulation, Poitiers University Hospital, 86,021 Poitiers, France. ^9^Pprime Institute UPR 3346, CNRS, ISAE-ENSMA, University of Poitiers, 86,360 Chasseneuil-du-Poitou, France. ^10^Department of Radiology, Universitair Ziekenhuis Brussel, Laarbeeklaan 101, 1090 Brussels, Belgium.
Name and contact information for the trial sponsor {5b}	Vrije Universiteit Brussel, Maarten Moens, maarten.TA.moens@vub.be
Role of sponsor {5c}	The funders had no influence on the research reported in this paper.

## Introduction

### Background and rationale {6a}

Patients suffering from persistent or recurring low back pain despite having undergone lumbosacral spine surgery, sometimes associated with referred or radiating leg pain [1], are denoted as suffering from chronic pain after spinal surgery or persistent spinal pain syndrome type 2 (PSPS-T2) [2]. This heterogeneous group of patients with PSPS-T2, based on aetiology and multilevel complaints (e.g. pain intensity, limited functionality), ends up with chronic pain and disability, which severely impacts individual’s health-related quality of life and patient participation, has a high psychological morbidity and poses a high socio-economic burden to society [3, 4].

Treatment of PSPS-T2 is complex as the condition involves neuropathic and/or nociceptive elements [5], whereby spinal cord stimulation (SCS) may serve as preferred option in case conservative treatment is not successful [6]. SCS is a type of neuromodulation that involves the implantation of an epidural electrode, connected through extensions with a subcutaneous implanted pulse generator [7]. Electrical pulses at different frequencies are generated and delivered to the spinal cord to elicit paresthesia in the painful area [8]. The efficacy of SCS in patients with PSPS-T2 has been demonstrated in several randomised controlled trials, clearly pointing out the value of SCS in comparison to conventional management. In the PROCESS trial [9], 100 patients with PSPS-T2 were randomised to either SCS with best medical treatment (BMT) or BMT alone. The primary outcome, i.e. the proportion of patients achieving 50% or more pain relief in the legs at 6 months, was obtained by 48% and 9% in the SCS + BMT and BMT, respectively [10]. In 2019, the PROMISE trial was published in which 218 PSPS-T2 patients with predominant low back pain were randomised to SCS + BMT versus BMT. In this trial, the primary outcome, i.e. the proportion of patients achieving ≥ 50% pain relief in the back at 6 months, was obtained by 13.6% and 4.6% in the SCS + BMT and BMT, respectively [11].

Since its first application in 1967, different stimulation parameters have been investigated to optimise the therapeutic efficacy of SCS [12]. The initial success of SCS was based on obtaining an adequate coverage over the patients’ pain areas with paresthesia by manipulating the pulse width or amplitude in a therapeutic way [13]. However, this traditional manner of stimulation has important limitations, as not every patient tolerates the sensation of paresthesias, leading to failure of SCS [14, 15]. In the last decade, the focus shifted towards the application of paresthesia-free paradigms, whereby superiority of the newer SCS designs was claimed in comparison to standard SCS [16, 17]. Nevertheless, all these studies explored a new subthreshold SCS paradigm compared to standard SCS, thereby relying on previous literature that standard SCS is better than BMT to obtain pain relief, and consequently fall back upon an indirect comparison. Up till now, there is no direct head-to-head evidence that new subthreshold SCS stimulation paradigms, i.e. those that are not providing paresthesia, are better than BMT for patients with PSPS-T2. Moreover, the primary outcome measurement of all previously mentioned trials was to obtain a pain intensity reduction, which entails a unidimensional primary outcome measure. This is in strong contrast with the recently proposed initiatives to shift towards a more comprehensive outcome measure, i.e. evaluating patients from a holistic point of view, taking functioning, medication use, quality of life, satisfaction and other measures into account besides pain intensity scores [18–23]. It thus becomes clear that only focusing on a pain intensity reporting is a serious denial of the complexity of pain [24].

### Objectives {7}

Given the substantial socioeconomic impact of SCS implantations, given that currently no direct high-quality evidence is available for subthreshold SCS versus BMT and given the lack of a holistic outcome to guide the treatment choice between subthreshold SCS versus BMT in patients with PSPS-T2, we here propose a randomised controlled trial to answer this question.

The primary scientific objective is to examine whether subthreshold SCS, compared to BMT, provided to patients with PSPS-T2 results in a different proportion of clinical holistic responders at 6 months. The secondary objective of the study is to examine if subthreshold SCS compared with BMT is different in improving patients’ individual competencies for self-management, increasing the likelihood to return to work, work status and healthcare expenditure, improving pain relief, obtaining pain medication reduction, decreasing anxiety and depression, increasing quality of life and decreasing disability.

### Trial design {8}

TRADITION is a two-arm, cross-over multicentre randomised controlled trial to evaluate whether subthreshold SCS results in more clinical holistic responders in PSPS-T2 patients compared with BMT. This research is a post-market clinical follow-up (PMCF) investigation [25]. Patients will be randomised (1:1) to (a) BMT or (b) subthreshold SCS. After the assessment at 6 months, patients are allowed to switch groups in case of unsatisfactory responses. Patients will have a follow-up period until 12 months after the start of the intervention.

## Methods: participants, interventions and outcomes

### Study setting {9}

The study will be conducted in one academic hospital (Universitair Ziekenhuis Brussel) and four regional (non-academic) hospitals (AZ Turnhout, AZ Delta, Jessa Ziekenhuis and AZ Sint-Maarten). All study sites are located in Belgium. Details on study sites can be found at ClinicalTrials.gov with Identifier: NCT05169047 registered on December 23, 2021.

## Participants

### Eligibility criteria {10}

This study will focus on patients with PSPS-T2, defined as patients suffering from neuropathic pain of radicular origin with pain in the lower back and/or leg(s), of an intensity of at least 4/10 on the numeric rating scale, for a period of at least 6 months after a minimum of one anatomically successful spinal surgery and refractory to conservative treatment (according to Belgian reimbursement rules from January 1, 2018). All patients need to be eligible for subthreshold SCS implantation to be eligible for participation in the study. Patients need to be at least 18 years old.

Exclusion criteria are an expected inability to operate the SCS system, an existing pregnancy, evidence of an active psychiatric disorder, or suffering from another chronic illness characterised by chronic generalised widespread pain (e.g. rheumatoid arthritis, fibromyalgia, chronic fatigue syndrome, scleroderma).

### Who will take informed consent? {26a}

The treating neurosurgeon or anaesthesiologist (local principal investigator or his/her designee) will inform eligible patients about the project. Thereafter, an investigator of TRADITION will contact the patients by telephone to further inform eligible patients about the project. In case they provide oral consent, they will be screened for in- and exclusion criteria as listed above during this telephone call. Patients who are eligible for participation (based on the telephone interview) and are willing to participate will receive detailed oral and written information about the study and have the opportunity to ask questions. Subsequently, they will be asked to provide written informed consent before participation.

### Additional consent provisions for collection and use of participant data and biological specimens {26b}

No biological samples will be obtained throughout this study. There are no planned ancillary studies involving the collection or derivation of data for purposes that are separate from the main trial.

## Interventions

### Explanation for the choice of comparators {6b}

In clinical practice, the number of physicians that is still programming their patients on standard SCS is rather limited. Similarly, analysis of patients’ preferences revealed a clear trend toward paresthesia-free SCS [26]. Based on qualitative research in patients with PSPS-T2 towards goal identification with SCS, it became clear that besides pain relief, patients aimed to improve walking (100%), sitting (73%), driving a car (67%), feeling happy (73%) and regaining a social life (73%) which emphasises the importance of broad outcome measurements in chronic pain management [27, 28] and consequently a suitable composite measure.

### Intervention description {11a}

#### Control intervention: best medical treatment (BMT)

For each patient who is randomised to BMT, an optimal individual treatment plan will be developed by the treating physician. BMT can include oral medications (i.e. opioid, non-steroidal anti-inflammatory drug, antidepressant, anticonvulsant/antiepileptic and other analgesic therapies), nerve blocks, epidural adhesiolysis, neurotomies, physical and psychological rehabilitative therapy and/or chiropractic care [10, 11]. The protocol specifically excludes other invasive therapy, such as spinal surgery or implantation of an intrathecal drug delivery system. Starting from randomisation, BMT is actively managed and according to local clinical practice, where the treatment plan is optimised at each visit. The number of visits is according to standard clinical practice of the participating centres for PSPS-T2 patients. All interventions are documented in diaries for healthcare utilisation by the patients.

#### Experimental intervention: subthreshold SCS

Participants randomly allocated to subthreshold SCS will undergo two surgical interventions to implant the neurostimulator. According to the Belgian reimbursement rules, all patients first need a SCS-trial period of 3 weeks with an external neurostimulator. After a successful trial according to Belgian legislation, i.e. pain reduction of at least 50% and reduction in medication use of at least 50%, the external neurostimulator will be replaced by an internal pulse generator (IPG). The implanting physician is free to choose the type of IPG that will be implanted.

SCS will be programmed at subthreshold stimulation, next to the conventional medical treatment that each patient will receive. As such, this intervention is similar to the BMT group with the addition of subthreshold SCS. Several subthreshold SCS stimulation types are provided such as high frequency SCS, burst SCS, high-dose SCS and differential target multiplexed SCS. For all stimulation types, patients do no longer feel paresthesia. Programming to subthreshold SCS will be performed on the day of IPG implantation.

#### Criteria for discontinuing or modifying allocated interventions {11b}

The participants can withdraw from the study at any time. The intervention can be discontinued in case the treating physician considers this appropriate concerning patient safety.

#### Strategies to improve adherence to interventions {11c}

At the outcome assessments, patients will be asked to indicate what type of interventions they followed during the past period. No other formal verification will be performed to control adherence to BMT or SCS.

#### Relevant concomitant care permitted or prohibited during the trial {11d}

Patients randomised to BMT will be asked not to start the trial SCS procedure during the first 6 months. By imposing these restrictions, adherence to the protocol can be guaranteed, whereafter patients can cross-over to the other group.

#### Provisions for post-trial care {30}

This study does not provide post-trial care. There is no anticipated harm for trial participation and consequently no compensation for anticipated harm.

### Outcomes {12}

#### Primary outcome

The primary outcome measure is the proportion of clinical holistic responders at 6 months. This outcome measure consists of a combination of 5 questionnaires whereby all criteria need to be fulfilled to be considered a clinical holistic responder. For questionnaires where a minimal clinical important difference (MCID) is available, patients should reach this cut-off value. A patient is considered a responder if all of the following criteria are fulfilled:

- 30% VAS pain intensity reduction for overall pain compared to baseline [29]
- 41% reduction in pain medication use compared to baseline [30]
- 30% improvement on Oswestry disability index (ODI) compared to baseline [29]
- MCID on EuroQol (EQ-5D)-5L compared to baseline [31]
- Minimally improved, much improved or very much improved on Patient Global Impression of Change (PGIC) [32]

#### Secondary outcomes

The visual analogue scale (VAS; 100 mm) will be used for the assessment of overall pain, defined as a combination of back and leg pain (but not pain from other body parts). The VAS pain score is believed to be reliable, valid and sensitive to change [33, 34].

Medication use (type of medication, dosage and frequency) will be recorded at each visit. Medication will be converted to one score with the Medication Quantification Scale III (MQS). The MQS is designed to quantify pain medication regimens in a wide variety of pain conditions [35]. It provides a numerical output that represents the negative impact of each medication [36]. For each medication, a MQS score is calculated by multiplying a detriment weight for a given pharmacologic class with a score for dosage [37]. Medication is subdivided into five classes: non-steroidal anti-inflammatory drugs (NSAIDs), muscle relaxants, neuropathic pain medications (antidepressants and anticonvulsants), benzodiazepines and opioids. All calculated values are summed to obtain a total MQS score. The cut-off value of 41% reduction in pain medication is calculated in this population [30].

The Oswestry disability index (ODI) is used to measure functional disability due to abnormalities of the spine [38, 39]. It contains ten topics for which each topic is scored on a scale from zero (no disability) to five (maximum disability possible). A total score of 100% indicates total disability, and higher scores correspond to more disability.

Health-related quality of life is assessed by the EuroQol with five dimensions and 5 levels (EQ-5D-5L) [40]. Patients subjectively tick the box with the most appropriate statement in each of the 5 dimensions (mobility, self-care, usual activities, pain/discomfort and anxiety/depression). The EQ-5D-5L index scores range from − 0.42 to 1, with 0 and 1 corresponding respectively to death and full health, based on preference-weighted health state classification algorithms [41]. Belgian population norms are available for the EQ5D-5L [42].

Patients will be asked to quantify the impression of change after treatment using the Patient Global Impression of Change scale (PGIC). The PGIC consist of a seven-point Likert scale asking the respondent to rate the overall level of improvement since the start of the treatment as “very much improved”, “much improved”, “minimally improved”, “no change”, “minimally worse”, “much worse” and “very much worse”[43].

A self-constructed questionnaire from the public health department of the VUB, specifically developed for a previous study evaluating return to work after spine surgery [44, 45] will be used to evaluate the work status. At baseline, current work status, job type, job description and work regime (e.g. full-time, part-time) will be asked. At the follow-up visits, patients will need to fill in whether they already resumed professional activities and, if yes, since when and to what extent. Additionally, patients will receive the opportunity to indicate whether a job change was needed or whether their job content needed to be changed to enable effective work resumption.

Patient Activation Measure-13 (PAM) is a 13-item instrument which assesses self-reported behaviour, knowledge and confidence for self-management of one’s health. PAM- 13 has proven to be a reliable instrument to measure patient activation and self-management [46, 47]. Patients will be divided in 4 levels, going from disengaged with lack of confidence (level 1) to individuals who maintain their healthy lifestyle and feel confident about their health (level 4).

The hospital Anxiety and Depression Scale (HADS) aims to measure symptoms of anxiety and depression and consists of 14 items: seven items for the anxiety subscale (HADS Anxiety) and seven for the depression subscale (HADS Depression). Each item is scored on a response-scale with four alternatives ranging between 0 and 3. After adjusting for six items that are reversed scored, all responses are summed to obtain the two subscales. Recommended cut-off scores are 8–10 for doubtful cases and ≥ 11 for definite cases [48]. HADS was found to perform well in assessing the symptom severity of anxiety disorders and depression in both somatic, psychiatric and primary care patients and in the general population [49].

Health seeking behaviour will be evaluated by self-reporting methods (diaries and questionnaires) [44]. Hence, healthcare expenditure includes hospital stays and any kind of treatments and consultations (e.g. pain killers, physiotherapy, psychotherapy) multiplied by their respective unit costs, derived from National Tariffs lists.

**Fig. 1:**
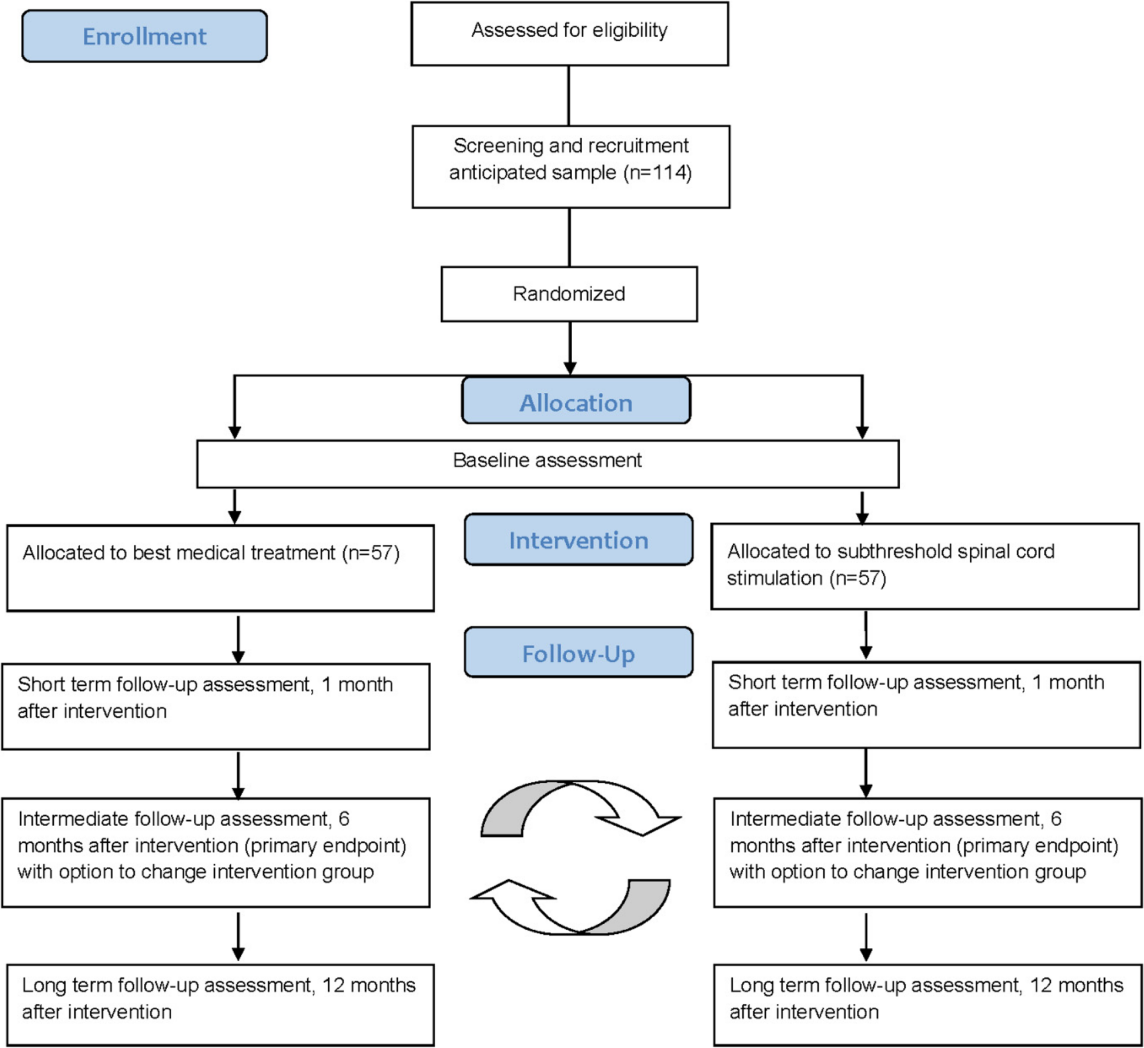
Project flowchart. Participant timeline. n, number

At baseline, all outcome measurements will be evaluated except for the impression of change and healthcare expenditure. Follow-up assessments will be performed one month after the start of the individualized best conservative treatment (BMT group) or 1 month after IPG implantation (SCS group), 6 months after start of the intervention (intermediate effects and primary endpoint) and 12 months (long-term effects) after intervention initiation. At 1 month and 12 months, outcome measurements can be completed without an additional hospital visit. Patients will receive a link towards the questionnaires to complete them online. At the 6-month follow-up, patients will complete the questionnaires at the hospital. After filling in the questionnaires at the 6-month follow-up visit, patients could change treatment groups (in both directions). This decision will be a shared decision between the patient and treating physician in case the randomised intervention did not provided enough pain relief. The cross-over will be mainly performed from BMT towards subthreshold SCS, according to study results obtained by Kumar et al. who evaluated standard SCS compared to BMT [10]. Figure 1 presents the project flowchart.

##### Participant timeline {13}

**Fig. 2:**
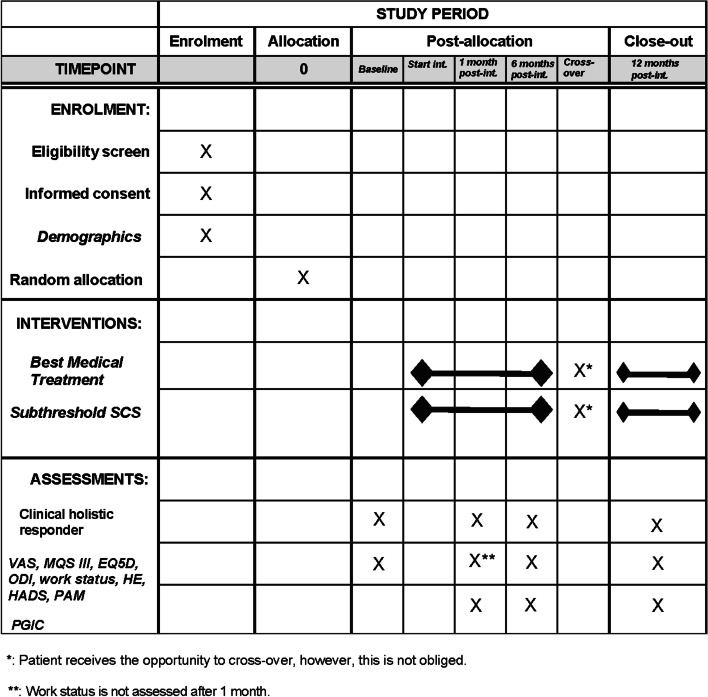
Participant timeline (SPIRIT). EQ5D, EuroQol with five dimensions and 5 levels; HADS, Hospital Anxiety and Depression Scale; HE, healthcare expenditure; int, intervention; MQS III, Medication Quantification Scale Version III; ODI, Oswestry disability index; PAM, Patient Activation Measure-13; PGIC, Patient Global Impression of Change; post-int, post-intervention; SCS, spinal cord stimulation; VAS, visual analogue scale

The participant timeline for TRADITION is presented in Fig. 2.

#### Sample size {14}

Sample size calculation was performed using the formula for large sample tests for proportions [50] and more specifically to test for equality between the proportion of clinical holistic responders in both interventions. The estimated proportion of clinical holistic responders is estimated at 47.8% in the subthreshold SCS group [51] and assumed to be 12.6% in the BMT group. The latter is an assumption based on the mean of the difference in primary outcome measures from the PROCESS and PROMISE trials at 6 months since no values for clinical holistic responders in BMT are available yet [10, 11]. By assuming an equal allocation, a sample size of 114 participants is needed with 57 participants in each intervention group. This sample size calculation accounts for a 20% loss to follow-up after 6 months and a 12% trial failure rate [51]. Calculations were performed for achieving an 80% power at the 5% level of significance.

#### Recruitment {15}

Patient recruitment will take place in several centres in Belgium: Universitair Ziekenhuis Brussel, AZ Turnhout, AZ Delta, AZ Sint-Maarten and Jessa Ziekenhuis. Patients were recruited from January 14, 2022, onwards. Depending on the rate of inclusion, we will contact other centres as well. Recruitment is expected to last for 2 years with an estimated rate of recruitment of 1 to 2 patients per month in each centre. Treating neurosurgeons or anaesthesiologists (regional coordinating investigator or his/her designee) will inform eligible patients about the project when patients are eligible for a treatment trajectory with subthreshold SCS.

### Assignment of interventions: allocation

#### Sequence generation {16a}

Patients will be randomly allocated (1:1 ratio) to BMT or subthreshold SCS using a computer-generated random list (Sealed Envelope Ltd., available from: https://www.sealedenvelope.com/simple-randomiser/v1/lists [Accessed 28 Dec. 2021]). To reduce predictability of a random sequence, a blocking procedure will be used including random block sizes of 2 and 4 patients. Randomisation will be stratified by investigational site.

#### Concealment mechanism {16b}

Only an unblinded researcher will have direct access to the randomisation list (Excel-file, which will be inaccessible for the outcome assessor and statistician).

#### Implementation {16c}

The unblinded researcher will generate the allocation sequence and is responsible for informing the patients and physicians to which group the patients are randomised.

### Assignment of interventions: blinding

#### Who will be blinded {17a}

Due to the nature of the treatments, the treating physicians and patients could not be blinded to the treatment group. The statistician and outcome assessor will be blinded to group allocation. With regard to this, patients will be asked not to communicate with the assessors about the intervention received.

#### Procedure for unblinding if needed {17b}

The statistician and outcome assessors will be remained blinded during the trial.

### Data collection and management

#### Plans for assessment and collection of outcomes {18a}

At baseline, all outcome measurements will be evaluated except for the impression of change and healthcare expenditure. For both groups, follow-up assessments will take place after 1 month, 6 months (primary endpoint) and 12 months. All outcome measurements will be collected through an online platform (Qualtrics), except for healthcare expenditure that is collected through a diary (up to 6 months) and at 12 months with a questionnaire. At 1 month and 12 months, outcome measurements can be completed without an additional hospital visit. Patients will receive a link towards the questionnaires to complete them online. In case patients do not have access to online resources, patients can fill in the questionnaires at the hospital, where the outcome assessor will be blinded to group allocation. With regard to this, patients will be asked not to communicate with the assessor about the intervention they received. At the 6-month follow-up, patients will complete the questionnaires at the hospital, whereafter they may cross-over towards the other intervention group in shared decision with the treating physician.

#### Plans to promote participant retention and complete follow-up {18b}

Patients receive a reminder of upcoming appointments and follow-up phone calls to promote participant retention and completion. For two follow-up visits, data will be collected remotely to minimise the burden for patients.

#### Data management {19}

Data will be collected via web-based self-reported questionnaires. To avoid missing data, an error will appear in case a question is not filled in. Collected data will include answers to validated questionnaires related to the patients’ overall pain intensity, disability, medication use, health-related quality of life, global impression of change, work status, competencies for self-management, healthcare expenditure, anxiety and depression. In addition, patients will be asked about the number of previous spine surgeries and general demographic data. Written informed consent of the patients will be collected and provides the basis for the legal ground for the data management.

During the research, the unblinded researcher is responsible for data management and storage. Following the research, the PI will be fully responsible for data management and storage. During the research, all obtained data will be stored on a dedicated page on Vrije Universiteit Brussel SharePoint (system-encrypted) with access limited to the TRADITION researchers. A back-up will be foreseen on a secure external hard drive. Following the research, all data will be relocated to the Vrije Universiteit Brussel Archive where it will be archived for 25 years. All possible personal identifiable data (vide infra) will be removed from the archived data.

Personal data will be processed in accordance with the ongoing European Union’s Data Protection Directive and regulation, the relevant Belgian legislation concerning data protection of July 30, 2018, and good clinical practice. As we collect personal identifiable data, following steps are taken to limit unauthorised access. Informed consents will be preserved at a secure location at the Vrije Universiteit Brussel. Qualtrics (Qualtrics, Provo, UT) will be used for data collection to improve data protection as responses to questionnaires will only be accessible to the investigator. Collected data will be password protected. Personal identifiable and clinical trial data will be separated, with the latter receiving a unique participant ID. Access to informed consents, personal identifiable data and the link with the participant ID will be restricted to the researchers and stored separately from the trial data. Eventual further dissemination of data will only occur in a pseudonymised or aggregated way.

The Vrije Universiteit Brussel supports the FOSB metadata standard (= dataset metadata schema defined by the Flemish Open Science Board) which can be mapped to the international DataCite metadata schema. At the project level, the general information (title, investigators, aim, objectives, concepts, hypotheses, funder), protocol, sampling procedure, instruments, hardware and software used to collect data, data handling log, accessibility of the data and data manipulations will be provided in research plans and publications. At the database level, an inventory of the files will be provided in a read-me file. At the data level, a codebook will be provided on how to handle quantitative variables together with the scripts to analyse the data.

#### Confidentiality {27}

Participant identification codes will be used to link data to patients. The file containing the linking between participant numbers and personal data (i.e. key) will be managed by the researchers and will be locked for access by others. As an additional security measure, the file linking the pseudonymisation to the original direct identifiers will be encrypted before it is uploaded to SharePoint.

#### Plans for collection, laboratory evaluation and storage of biological specimens for genetic or molecular analysis in this trial/future use {33}

No biological specimens will be obtained during the conduct of the trial.

### Statistical methods

#### Statistical methods for primary and secondary outcomes {20a}

Considering the longitudinal data, a generalised estimating equation will be constructed to evaluate and compare therapy effects. The primary outcome will be analysed as the proportion of clinical holistic responders from baseline to 6 months (taking into account the longitudinal nature of the trial) between both groups. Baseline variables will be used as covariates. For secondary outcome measurements, a similar strategy will be applied with longitudinal mixed models. This longitudinal data analysis approach will also be applied for 12 months data. Statistical as well as clinical significant differences will be defined at alpha < 0.05. Furthermore, based on the baseline data, we will determine predictive factors and which subgroup of patients will benefit the most of the treatment. All analysis will be performed in SAS or R.

#### Interim analyses {21b}

No interim analyses will be conducted as we do not foresee any potentially serious outcomes.

#### Methods for additional analyses (e.g. subgroup analyses) {20b}

Baseline data will provide cross-sectional results on work status, pain intensity, quality of life, disability, medication use and self-management for the complete PSPS-T2 group and comparisons between possible subgroups. Furthermore, correlation analyses will be performed in order to unravel correlations between the different outcome measures in patients with PSPS-T2, eligible for SCS. Correlation analysis will be performed with Pearson correlation coefficients if the assumption of a linear relation between two variables is met; otherwise, Spearman correlation coefficients will be calculated and tested at alpha < 0.05.

A within trial economic evaluation will be conducted. All costs of all patients will be considered, for the time horizon starting from start of the allocated intervention until the 6-month follow-up period. Intervention costs will be based on the study notes documenting the duration of each session per patient. The valuation of resource use is based on national tariffs. Health outcomes will be expressed in two ways: (1) percentage holistic responders, which is the primary outcome in this trial, and (2) utility using health state values from the general public. The overall result is expressed in an incremental cost effectiveness ratio (ICER, i.e. incremental cost divided by the percentage increment in holistic responders and incremental cost divided by the incremental QALY gained). Differences in cost between both groups will be analysed using generalised linear models. The point estimates of incremental costs and increment health benefits (deterministic analyses) are subject to uncertainty which will be addressed in probabilistic analyses [52]. We will apply nonparametric bootstrapping to test for statistical differences in costs and health benefits to investigate the uncertainty around these outcomes and summarised in cost-effectiveness acceptability curves indicating the likelihood of the intervention to be cost-effective at any willingness-to-pay threshold. Reporting on the results of the health economic evaluation will be in line with the CHEERS II guidelines [53]. Besides the within-trial health economic evaluation, a model-based evaluation will be conducted in order to estimate the expected costs and health outcomes in both intervention groups beyond the follow-up period of the trial. A Markov-model will be developed compliant to the commonly used guidelines [54].

#### Methods in analysis to handle protocol non-adherence and any statistical methods to handle missing data {20c}

Both intention-to-treat as well as per protocol analyses will be conducted to determine whether both definitions of the population will point towards similar results, which could improve robustness of the results. At first, analysis will be performed on data as observed. Hence, intermittent missing is not expected to substantially bias the results. To address possible informative drop-outs, a sensitivity analysis will be performed using multiple imputation, including all available information on background characteristics and outcome. All analysis will be performed in SAS or R.

#### Plans to give access to the full protocol, participant level-data and statistical code {31c}

After finalising the project, access restrictions will be applied to the pseudonymised data and will be specified in a data use agreement containing following elements: evaluation of the re-use request by ethical committee, non-disclosure agreement and warranties for safely storage of data.

### Oversight and monitoring

#### Composition of the coordinating centre and trial steering committee {5d}

The steering committee is the main decision-making and steering body of the project. The steering committee consists out of M.M, K.P. and L.G. The steering committee organised a kick-off meeting at the start of the project to establish common working procedures. The main tasks of the steering committee are as follows: (1) agree on common working procedures and management policies, (2) monitor overall progress and follow-up of deliverables, (3) decisions on major changes to the work programme and (4) conflict handling. The steering committee is also responsible for assuring the quality of the workflow and project implementation, considering the available resources. The steering committee will further assemble meetings at least every 6 months. Additional teleconferences can be organised ad hoc in case of urgent issues.

The local principal investigators in the recruiting centres are responsible for patient recruitment and form the TRADITION consortium, together with the steering committee members.

The valorisation board, in which relevant stakeholders take part, will be asked to actively contribute to implementation of the study findings, and any difficulties experienced during the implementation process will be discussed. The researchers will support the stakeholders with the implementation process. In addition, the valorisation board will prepare and guide the full utilisation process in the period following the completion of the research project (after-trajectory), together with the steering committee.

#### Composition of the data monitoring committee, its role and reporting structure {21a}

The unblinded researcher at Vrije Universiteit Brussel will regularly monitor data that are entered in Qualtrics. This researcher is independent from the funder of this study and has no competing interests.

#### Adverse event reporting and harms {22}

All adverse events (AEs) reported spontaneously by the patient or observed by the researcher will be recorded. Serious adverse events (SAEs) will be reported to the (local) principal investigators as soon as possible, who will be responsible for informing the ethics committee.

#### Frequency and plans for auditing trial conduct {23}

The steering committee will submit a summary of the progress of the trial to the central ethics committee once a year. Information will be provided on the date of inclusion of the first patient, numbers of patients included and numbers of patients that have completed the trial, serious adverse events/serious adverse reactions, other problems and amendments. There is no planned on-site auditing of the trial. However, to ensure compliance with relevant regulations, an independent quality assurance representative may review this study. This implies that auditors will have the right to audit the site(s) at any time during and/or after completion of the study and will have access to the data generated during the clinical investigation, source documents and patient’s files.

#### Plans for communicating important protocol amendments to relevant parties (e.g. trial participants, ethical committees) {25}

All protocol amendments will be approved of by the ethics committee prior to implementation. If relevant, patients will be informed of protocol modifications.

#### Dissemination plans {31a}

The primary goal of this study is to provide head-to-head evidence for subthreshold SCS compared to BMT with a relevant outcome measure, namely a clinical holistic responder. All stakeholders will be contacted during the execution of the project to further introduce this concept of a composite measure in relation to PSPS-T2 patients. Regular updates will be provided to all stakeholders to keep everyone informed, involved and motivated for this project. The definition of ‘clinical holistic responder’ is not a static feature, we will organise discussions with peers to keep fine-tuning this concept with relevant information. Further, we will communicate findings of this project via the publication of scientific manuscripts and presentations on national and international symposia, as well as through the social media (STIMULUS & UMCOR social media accounts). Furthermore, we will write a summary of the main study findings in layman’s terms for patients’ organisations and charities. Additionally, social instances will be contacted and presentations will be given to make them aware of the study findings.

## Discussion

Currently, the field of neuromodulation is expanding enormously whereby the last decade is characterised by refinements and innovations in the form of novel stimulation waveforms, new anatomical targets and novel feedback-loop mechanisms [55–58]. Innovations and technical improvements are intertwingled with ethical considerations, whereby new stimulation paradigms undoubtedly influence the neuromodulation landscape and the day-to-day decisions of implanting physicians to choose for specific devices and stimulation paradigms [59]. Despite the indirect influence on decision-making of physicians, and the substantial socioeconomic impact of SCS implantations, no direct high-quality evidence is available to guide the treatment choice between subthreshold SCS versus BMT in patients with PSPS-T2. Therefore, TRADITION will not contribute to the rat race of innovations and instead take one step back to evaluate a ‘*basic*’ clinical research question with a relevant composite measure.

### Trial status

Recruitment has started in January 2022 and will be ongoing until 114 patients are included in the study (expected end date December 2023). The current protocol is version 2 December 2021.

## Data Availability

The steering committee has access to the pseudonymised final trial dataset. Any materials to support the protocol can be supplied on motivated request, under the condition that the current study is not compromised.

## Abbreviations

BMT: Best medical treatment
EQ5D-5L: EuroQol with five dimensions and 5 levels
HADS: Hospital Anxiety and Depression Scale
HE: Healthcare expenditure
IPG: Implantable pulse generator
ODI: Oswestry disability index
PSPS-T2: Persistent spinal pain syndrome type II
PAM: Patient Activation Measure-13
SCS: Spinal cord stimulation
VAS: Visual analogue scale
